# α-Difluoromethylornithine-Induced Cytostasis is Reversed by Exogenous Polyamines, Not by Thymidine Supplementation

**DOI:** 10.3390/biom11050707

**Published:** 2021-05-10

**Authors:** Mervi T. Hyvönen, Maxim Khomutov, Jouko Vepsäläinen, Alex R. Khomutov, Tuomo A. Keinänen

**Affiliations:** 1 Kuopio Campus, School of Pharmacy, Biocenter Kuopio, University of Eastern Finland, Yliopistonranta 1C, 70210 Kuopio, Finland; jouko.vepsalainen@uef.fi (J.V.); tuomo.keinanen@uef.fi (T.A.K.); 2 Engelhardt Institute of Molecular Biology, Russian Academy of Sciences, Vavilov Street 32, 119991 Moscow, Russia; makhomutov@mail.ru (M.K.); alexkhom@list.ru (A.R.K.)

**Keywords:** polyamines, thymidine, α-difluoromethylornithine, proliferation, *S*-adenosyl-*L*-methionine

## Abstract

Polyamine spermidine is essential for the proliferation of eukaryotic cells. Administration of polyamine biosynthesis inhibitor α-difluoromethylornithine (DFMO) induces cytostasis that occurs in two phases; the early phase which can be reversed by spermidine, spermine, and some of their analogs, and the late phase which is characterized by practically complete depletion of cellular spermidine pool. The growth of cells at the late phase can be reversed by spermidine and by very few of its analogs, including (*S*)-1-methylspermidine. It was reported previously (Witherspoon et al. Cancer Discovery 3(9); 1072–81, 2013) that DFMO treatment leads to depletion of cellular thymidine pools, and that exogenous thymidine supplementation partially prevents DFMO-induced cytostasis without affecting intracellular polyamine pools in HT-29, SW480, and LoVo colorectal cancer cells. Here we show that thymidine did not prevent DFMO-induced cytostasis in DU145, LNCaP, MCF7, CaCo2, BT4C, SV40MES13, HepG2, HEK293, NIH3T3, ARPE19 or HT-29 cell lines, whereas administration of functionally active mimetic of spermidine, (*S*)-1-methylspermidine, did. Thus, the effect of thymidine seems to be specific only for certain cell lines. We conclude that decreased polyamine levels and possibly also distorted pools of folate-dependent metabolites mediate the anti-proliferative actions of DFMO. However, polyamines are necessary and sufficient to overcome DFMO-induced cytostasis, while thymidine is generally not.

## 1. Introduction

**Figure 1:**
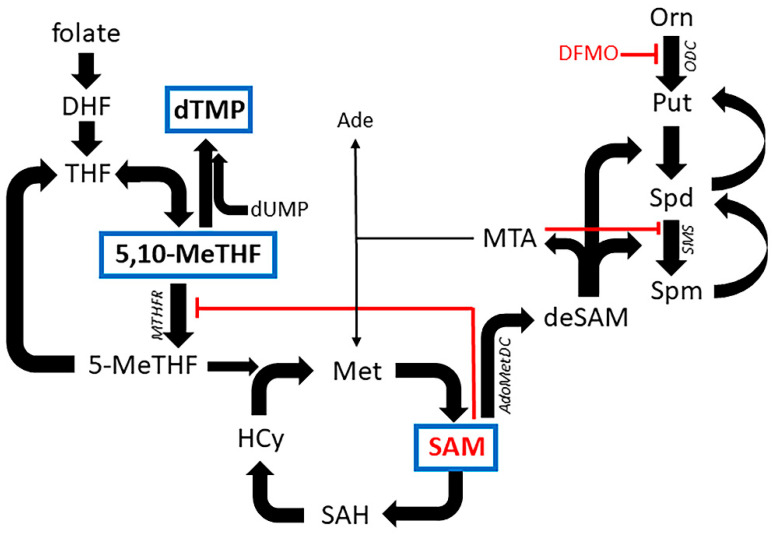
Metabolic pathways connecting folate, *L*-methionine and polyamine cycles. 5,10-MeTHF, 5,10-methylenetetrahydrofolate; 5-MeTHF, 5-methyltetrahydrofolate; Ade, adenine; AdoMetDC, S-adenosyl-*L*-methionine decarboxylase; deSAM, decarboxylated *S*-adenosyl-*L*-methionine; DFMO, α-difluoromethylornithine; DHF, dihydrofolate; dTMP, thymidine-5′-monophosphate; dUMP, uridine-5′-monophosphate; THF, tetrahydrofolate; HCy, homocysteine; Met, *L*-methionine; MTHFR, methylene tetrahydrofolate reductase; ODC, ornithine decarboxylase; Orn, *L*-ornithine; Put, putrescine; SAM, *S*-adenosyl-*L*-methionine; SAH, S-adenosylhomocysteine; SMS, spermine synthase, Spd, spermidine; Spm, spermine.

The polyamines spermidine (Spd) and spermine (Spm) and their diamine precursor putrescine (Put) (Figure 1, Figure S1) are cationic regulators of many important cellular functions such as proliferation and differentiation [1]. Polyamine metabolism is intimately connected to methionine, folate, urea, and tricarboxylic acid cycles (Figure 1). Activation of polyamine biosynthesis is associated with carcinogenesis, whereas polyamine depletion leads to cytostasis or cytotoxicity. α-Difluoromethylornithine (DFMO, Eflornithine^®^, Figure S1) is an irreversible inhibitor of ornithine decarboxylase (ODC), the key polyamine biosynthetic enzyme. DFMO treatment causes reduction of intracellular Put and Spd levels, while its effect on Spm pools varies in between cell lines. We and others have shown that the treatment with DFMO leads to the cessation of cell growth in two distinct phases; the early phase, which can be reversed by the natural polyamines and many of their analogs, and the late phase, which can be reversed only by Spd and some of its functionally active mimetics such as (*S*)-1-methylspermidine (MeSpd, Figure S1) [2]. Spd functions as the sole known natural donor of the aminobutyl group in the synthesis of hypusine [*N*-ε-(4-amino-2-hydroxybutyl)-*L*-lysine]. Hypusine is a unique amino acid, formed to the ε-amino group of Lys-50 of eukaryotic initiation factor 5A (eIF5A) protein as a result of posttranslational modification [3]. Although initially named as an initiation factor, it is now known that hypusinated eIF5A also promotes translation elongation and translation of polyproline-rich proteins and that it is essential for the proliferation of eukaryotic cells.

DFMO is in clinical use against stage 2 African sleeping sickness (trypanosomiasis) and facial hirsutism, and it has been extensively tested as an anticancer agent [4,5]. Moreover, DFMO has advanced to cancer chemoprevention trials, as a single agent and in combination with anti-inflammatory drug sulindac or low polyamine content diet with promising results. In IIb/III clinical trial, high-risk colon carcinoma subjects treated with DFMO/sulindac for 3 years exhibited 70% and 92% reductions in the incidences of adenomas and advanced adenomas, respectively [6]. It was also shown that while the drug efficacy was the greatest in subjects with a low Spd/Spm ratio in rectal mucosa at the beginning of the trial, no relationship was found between DFMO/sulindac-induced changes in polyamine levels and drug efficacy [7]. In mouse studies, DFMO-treatment and ODC heterozygosity were both able to impair lymphomagenesis in Eμ-Myc transgenic mice, while only DFMO was effective in suppressing MYCN-driven neuroblastoma, suggesting that DFMO might target also additional factors than polyamines [8].

Previously it was shown that in addition to depleting polyamine pools, DFMO treatment led to a reduction of cellular nucleoside and nucleotide pools, especially that of thymidine-5′-monophosphate (dTMP) [9]. In addition, it was shown that exogenous thymidine supplementation could partially prevent DFMO-induced cytostasis without affecting the intracellular polyamine pools in HT-29, SW480, and LoVo colorectal cancer cells.

In the present study, we investigated whether such growth-supporting effect of thymidine is a general feature of other cell lines, or whether it is restricted to some specific cell lines, such as colon carcinoma cell lines. We found no evidence of the ability of thymidine to rescue DFMO-induced growth inhibition either in CaCo2 or HT-29 colon carcinoma cells or in any other of the eight tested cell lines.

## 2. Materials and Methods

### 2.1. Materials

(*S*)-1-MeSpd was synthesized as described previously [10]. DFMO was a kind gift from Prof. P.M. Woster (Medical University of South Carolina, Charleston, SC, USA). Put, Spd, Spm, aminoguanidine, thymidine, and *L*-methionine were purchased from Sigma-Aldrich (St.Louis, MO, USA). SAM was purchased from Shanghai Kinpharm Engineering (Shanghai, China). The cell lines DU145, LNCaP, MCF7, BT4C, CaCo2, SV40MES13, HEK293, and HepG2 were obtained from American Type Culture Collection (Manassas, VA, USA). ARPE-19 cell line was a kind gift from Prof. A. Kauppinen (School of Pharmacy, University of Eastern Finland, Kuopio, Finland). HT-29 cell line was kindly provided by the Institute of Public Health and Clinical Nutrition (University of Eastern Finland, Kuopio, Finland). High-glucose DMEM and *L*-glutamine were from Sigma-Aldrich, heat-inactivated fetal bovine serum from Gibco (Calsbad, CA, USA), and gentamycin from Lonza (Basel, Switzerland).

### 2.2. Cell Culture

The cells were cultured in high-glucose Dulbecco’s Modified Eagle’s Medium supplemented with 10% heat-inactivated fetal bovine serum, 2 mM *L*-glutamine, and 50 μg/mL gentamycin. The cells were incubated in a humidified atmosphere at +37 °C, 5 % CO_2_. The cells were grown on 96-well plates and treated with 1–1000 µM thymidine, 50–1000 μM *L*-methionine, 10–1000 μM SAM or 100 µM (*S*)-1-MeSpd in the absence or presence of 5 mM DFMO. For testing of the natural polyamines, 10 μM Put, Spd, or Spm was used with 1 mM aminoguanidine (prevents the degradation of polyamines by amine oxidases present in the fetal bovine serum). Cell proliferation was analyzed using resazurin staining [11]. The correlation between resazurin staining and sulforhodamine B staining (protein amount) was confirmed before assays (data not shown). LDH release was determined from the medium by measuring the decrease in NADH absorbance at 340 nm as described earlier [12]. The experiments were performed twice with 6 biological replicates each time.

### 2.3. Polyamine Analysis

The cells grown on 6-well plates were detached using a solution containing 0.25 % trypsin and 1 mM EDTA in PBS. The washed cell pellet was mixed with sulfosalicylic acid (5% (w/v) final concentration) containing 10 μM diaminoheptane as internal standard and analyzed with HPLC (Perkin Elmer series 200 (Waltham, MA, USA), equipped with Pickering PCX 5200 post-column derivatization unit (Mountain View, CA, USA) as described earlier [13]. The results were normalized to protein amount in the sample. Protein concentrations were measured from polyamine sample pellets dissolved in 0.1 M NaOH, using a Bio-Rad protein kit with dilutions of bovine serum albumin (Bio-Rad, Hercules, CA, USA) as standards. The experiments were performed once with 3 biological replicates.

### 2.4. Statistical Analysis

One-way ANOVA with Dunnett’s or Tuckey’s post hoc test was used for statistical analysis with the aid of software package GraphPad Prism v. 5.03.

## 3. Results

### 3.1. Effects of Thymidine and 1-MeSpd on Growth of DFMO-Treated Cells

**Figure 2:**
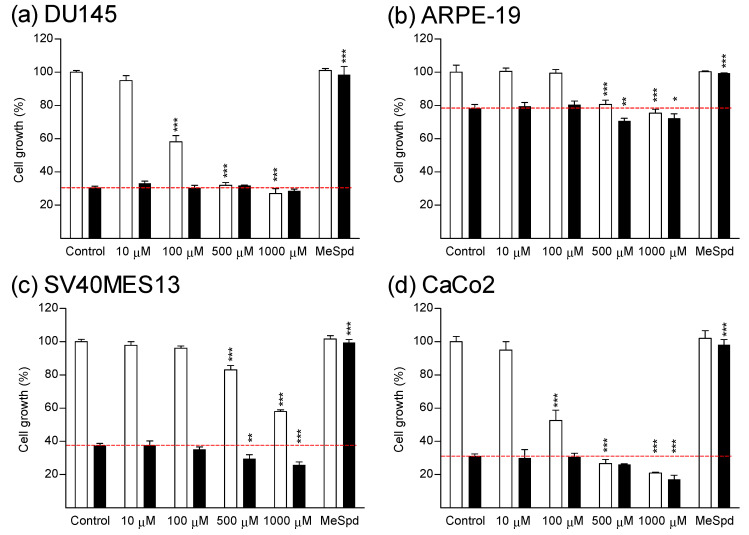
Effect of thymidine and (*S*)-1-MeSpd on the growth of various cell lines. (**a**) DU145, (**b**) ARPE-19, (**c**) SV40MES13 and (**d**) CaCo2 cells. The cells were treated with 10–1000 µM thymidine or (*S*)-1-MeSpd (100 µM) in the presence (black bars) or absence (white bars) of DFMO (5 mM) for 7 days (DU145 cells) or 4 days (other cell lines). Data are means ± SD, *n* = 6. *, **, and *** refer to statistical significance of *p* < 0.05, *p* < 0.01 and *p* < 0.001 as compared to control/DFMO groups, respectively. The red dotted line indicates the level of cell growth of DFMO-treated cells.

**Figure 3:**
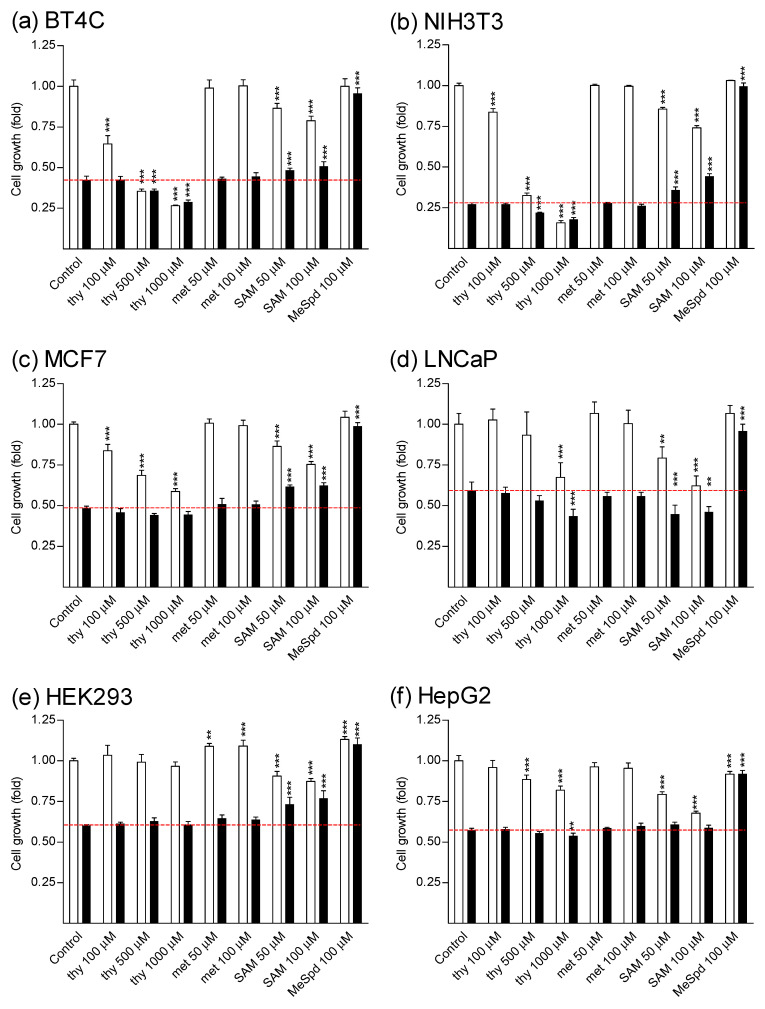
Effect of thymidine, *L*-methionine, SAM and (*S*)-1-MeSpd on growth of various cell lines. (**a**) BT4C, (**b**) NIH3T3, (**c**) MCF7, (**d**) LNCaP, (**e**) HEK293, and (**f**) HepG2 cells. The cells were treated with thymidine (thy, 100–1000 µM), *L*-methionine (met, 50–100 µM), SAM (50–100 µM) or (*S*)-1-MeSpd (MeSpd, 100 µM) in the presence (black bars) or absence (white bars) of 5 mM DFMO for 4 days. Data are means ± SD, *n* = 6. **, and *** refer to statistical significance of *p* < 0.01 and *p* < 0.001 as compared to control/DFMO groups, respectively. The red dotted line indicates the level of cell growth of DFMO-treated cells.

**Figure 4:**
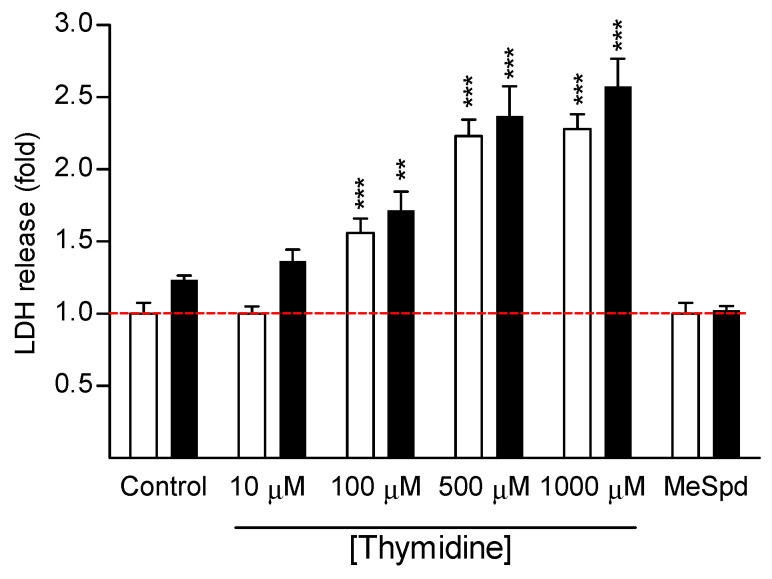
Cytotoxicity of thymidine to colon carcinoma CaCo2 cells. The cells were treated with 10–1000 µM thymidine or (*S*)-1-MeSpd (100 µM, MeSpd) in the presence (black bars) or absence (white bars) of 5 mM DFMO for 4 days. LDH release to the medium was measured. Data are means ± SD, *n* = 6. Statistical significance ** *p* < 0.001, *** *p* < 0.001 as compared to control/DFMO group. The red dotted line indicates the level of cell growth of control cells.

We first investigated whether thymidine could reverse DFMO-induced cytostasis in colorectal cancer cell line (CaCo2), or in various other cell lines. The selected cell lines were human hormone-insensitive prostate cancer cells (DU145) and human hormone-sensitive prostate cancer cells (LNCaP), human breast carcinoma cells (MCF7), immortalized mouse kidney mesangial cells (SV40MES13), human retinal pigment epithelial cells (ARPE-19), rat glioma cells (BT4C), human embryonic kidney cells (HEK293), human hepatoma cells (HepG2), and mouse embryonic fibroblasts (NIH3T3). As indicated in Figure 2 and Figure 3, DFMO (5 mM) inhibited the proliferation of all tested cell lines after 4–7 days of culture. Treatment of DU145 cells with either Put, Spd, or Spm (10 µM, with 1 mM aminoguanidine, serum amine oxidase inhibitor) fully reversed DFMO-induced cytostasis (Figure S2). Cytostasis was also completely or nearly completely reversed in all cell lines tested by (*S*)-1-MeSpd. (Figure 2 and Figure 3). We chose to use this analog instead of the natural polyamines because it is not metabolized by serum amine oxidases to toxic metabolites and therefore does not require the supplementation of aminoguanidine. Importantly, at a concentration range of 10–1000 µM thymidine was unable to restore the proliferation of any tested cell line. Thymidine itself was cytostatic at higher concentrations, and even cytotoxic in some cell lines, as evidenced by increased LDH release to medium in CaCo2 cell line (Figure 4). Changing fresh medium supplemented with thymidine daily did not improve its effect on cell growth (data not shown).

**Figure 5:**
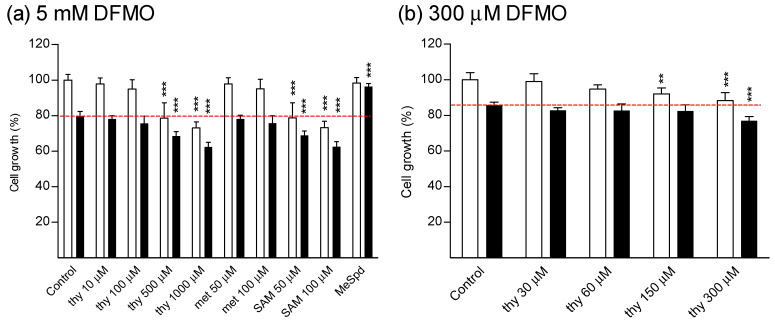
Effect of thymidine, *L*-methionine, SAM, and (*S*)-1-MeSpd on the growth of HT-29 colon carcinoma cells. (**a**) The cells were treated with thymidine (thy, 100–1000 µM), *L*-methionine (met, 50–100 µM), SAM (50–100 µM) or (*S*)-1-MeSpd (MeSpd, 100 µM) in the presence (black bars) or absence (white bars) of 5 mM DFMO for 4 days. (**b**) The cells were treated with thymidine (thy, 30–300 µM) in the presence (black bars) or absence (white bars) of 300 µM DFMO for 4 days. Data are means ± SD, *n* = 6. **, and *** refer to statistical significance of *p* < 0.01 and *p* < 0.001 as compared to control/DFMO groups, respectively. The red dotted line indicates the level of cell growth of DFMO-treated cells.

In contrast to earlier publication [9], we could not obtain any growth reversal with thymidine in DFMO-treated HT-29 colon carcinoma cells, even when using the same concentrations as in the previous study (300 μM DFMO and 30–300 μM thymidine) (Figure 5). It is noteworthy, that similar to the ARPE-19 cell line, HT-29 cells were very resistant to DFMO treatment, with only 20% growth inhibition after 4 days of culture in the presence of 5 mM DFMO.

### 3.2. Effects of L-Methionine and SAM on Growth of DFMO-Treated Cells

Treatment with *L*-methionine (50–100μM, Figure S1), was also ineffective in restoring cell growth of DFMO-treated cells, whereas some reversal was seen with treatment with *S*-adenosyl-*L*-methionine (SAM, 50–100 μM, Figure S1), the precursor of decarboxylated SAM, in some, but not in all tested cell lines (Figure 3, Figure 5). Increasing SAM concentration up to 500–1000 µM did not further increase the growth of DFMO-treated cells (data not shown). SAM alone displayed a concentration-dependent cytostatic effect (Figure 3, Figure 5, Figure S4), which could not be reversed by the addition of Put, Spd, or Spm (10 μM, in the presence of 1 mM aminoguanidine) (Figure S3). *L*-Methionine alone (50–100 μM) did not affect cell growth (Figure 3, Figure 5).

### 3.3. Effects of DFMO, Thymidine, L-Methionine and SAM on Polyamine Levels

**Figure 6:**
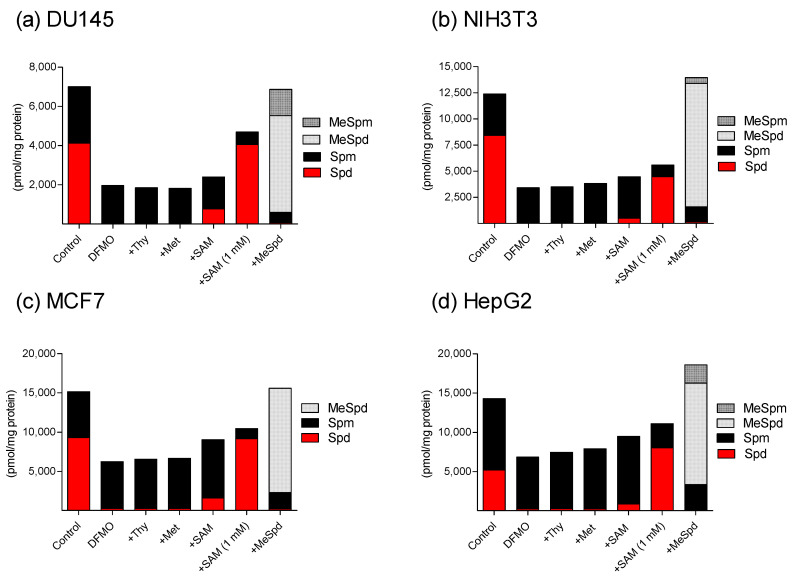
Polyamine levels in (**a**) DU145, (**b**) NIH3T3, (**c**) MCF7 and (**d**) HepG2 cells treated for 3 days with DFMO (5 mM) only (DFMO), or in combination of DFMO with thymidine (Thy, 100 μM), *L*-methionine (Met, 100 μM), SAM (100 μM or 1 mM), (*S*)-1-MeSpd (MeSpd, 100 μM), or without any additions (Control). Data are means, *n* = 3, standard deviation markers were omitted for clarity (<10 % for all). MeSpm, (*S*)-1-MeSpm.

We next selected DU145, NIH3T3, MCF7, and HepG2 cell lines for the analysis of polyamine levels. DFMO-treatment led to dramatic decreases in intracellular Spd and total polyamine (Spd+Spm) levels, which were not affected by thymidine or by *L*-methionine treatments (Figure 6). (*S*)-1-MeSpd treatment further decreased intracellular Spd and Spm levels, but the total polyamine level (Spd+Spm+MeSpd+MeSpm) was restored to control levels due to the high accumulation of the analog and its intracellularly converted metabolite, (*S*)-1-MeSpm. Interestingly, treatment with the combination of DFMO+SAM increased the amount of Spd and the total polyamine pool and decreased the amount of Spm (Figure 6). By contrast, treatment with SAM alone decreased the total polyamine pool along with the decreased amount of Spm (Figure S4).

## 4. Discussion

SAM is the major cellular methyl group donor, involved in methylation, transsulfuration, and aminopropylation reactions. Metabolism of SAM by S-adenosyl-*L*-methionine decarboxylase (AdoMetDC) yields decarboxylated SAM (deSAM), which is used as aminopropyl donor in the synthesis of Spd and Spm (Figure 1) [1]. The production of dTMP is partly controlled by the cellular level of SAM. SAM functions as an allosteric (noncompetitive) inhibitor of methylenetetrahydrofolate reductase (MTHFR), an enzyme that converts 5,10-methylenetetrahydrofolate to 5-methyltetrahydrofolate (Figure 1). It was hypothesized that decreased SAM level observed in DFMO-treated cells switches the utilization of 5,10-methylenetetrahydrofolate from maintenance of thymidine pools to support SAM regeneration by relieving the repression of MTHFR. Indeed, a marked decrease in nucleotide and nucleoside levels, which were restored to normal by supplementation of Put, was observed [9]. In agreement with these findings, we have also observed earlier that 3-day DFMO treatment of DU145 cells leads to over 10-fold increase in AdoMetDC activity and a subsequent appearance of decarboxylated SAM and ~30 % decrease in SAM level [14].

Our and others’ earlier work shows that DFMO-induced “early phase” of cytostasis can be reversed by the natural polyamines and many of their analogs, even those which are not hypusine precursors [2,15]. However, only Spd and some of its functionally active mimetics, such as (*S*)-1-MeSpd can reverse the “late phase” of cytostasis which is caused by depletion of hypusinated eIF5A. In DU145 cell culture supplemented with 5 mM DFMO, the “late phase” is achieved typically within a week [2,15,16], whereas more slowly proliferating cell lines generally require a longer time (unpublished observations). It was earlier shown that even a very small amount of Spd is enough to support hypusination of eIF5A, and thus, proliferation, at least in yeast [17]. In their work, Witherspoon et al. [9] used DFMO at 1–9 mM and thymidine at 1–10 mM for 7 days for LoVo and SV480 cell lines, and 300 μM DFMO and 30–300 µM thymidine for HT-29 cells. Although not investigated in that study, we assume that hypusinated eIF5A was not decreased under the “critical level” even at the highest tested dose of DFMO (9 mM) since they found that thymidine could still partially overcome DFMO-induced cytostasis.

Here we were unable to obtain any reversal of DFMO-induced early cytostasis by using thymidine supplementation in any of the tested cell lines, even with shorter incubation time (4 days, i.e., the “early phase” of cytostasis). Yet, the classical add-back experiments with natural polyamines and methylated Spd analog restored proliferation. These results indicate that reversal of DFMO-induced cytostasis by thymidine seems to be restricted only to some special cell types, such as SV480 and LoVo. It seems that it is not even specific for colon carcinoma cells in general, since we observed no growth-supporting effect of thymidine in CaCo2 colon carcinoma cells. Thymidine was even cytotoxic to CaCo2 cells, as indicated by increased LDH release (Figure 4). Here we could not reproduce the results obtained in the previous study [9] for HT-29 cell line with 300 μM DFMO and 30–300 μM thymidine (Figure 5). We have no solid explanations for this discrepancy why our HT-29 data are different from those obtained by [9]. We can just speculate on possible mutations in the used HT-29 cell lines, the use of different growth media, or differences in used serum batches.

Surprisingly, SAM supplementation could partially reverse DFMO-induced cytostasis in some cell lines, even though SAM alone was cytostatic. Examination of the intracellular polyamine pools revealed that the SAM+DFMO combination resulted in increased Spd and total polyamine pools and decreased Spm pool as compared to DFMO-treated cells. Therefore, increased total polyamine level could be responsible for the partial reversal of DFMO-induced cytostasis by SAM observed in some cell lines. In addition, some effects of SAM could be mediated by its degradation product 5′-deoxy-5′-methylthioadenosine (MTA). SAM is unstable in solution and is converted to MTA spontaneously, and intracellularly through the polyamine pathway (Figure 1). MTA has been shown to strongly inhibit spermine synthase (Ki~0.3 μM for bovine brain spermine synthase) [18] that likely explains the observed change in Spd/Spm ratio in DFMO+SAM-treated cells. It is noteworthy, that MTA itself is a known cytostatic/cytotoxic molecule, and its effect on cell proliferation is mediated through the relative rates of its production and degradation [19]. The finding that exogenous polyamine supplementation was unable to restore the growth of SAM-treated cells (Figure S3) indicates that SAM has also other targets than the polyamine pathway, such as the cellular methylome.

A sufficient pool of dTMP is essential for DNA synthesis, and thus, for cellular proliferation. While putrescine supplementation restores both polyamine and thymidine pools of DFMO-treated cells, thymidine supplementation restores only the dTMP pool [9]. Based on these results we conclude that decreased polyamine levels and possibly also distorted pools of folate-dependent metabolites mediate the anti-proliferative actions of the ODC inhibitor DFMO. However, polyamines are necessary and sufficient to overcome DFMO-induced cytostasis, while thymidine is generally not.

## Data Availability

Not applicable.

## Supplementary Materials

The following are available online at https://www.mdpi.com/article/10.3390/biom11050707/s1, Figure S1. Structures of various small molecules related to this work. Figure S2. Effect of natural polyamines on the growth of DFMO-treated DU145 cells. Figure S3. Effect of natural polyamines on the growth of SAM-treated DU145 cells. Figure S4. Effect of SAM on the growth and polyamine pools of NIH3T3 and HepG2 cells.
