# Supplementary material for: α-Difluoromethylornithine-Induced Cytostasis is Reversed by Exogenous Polyamines, Not by Thymidine Supplementation
Source: Biomolecules. 2021 May 10;11(5):707. doi: 10.3390/biom11050707 (PMC8151227; doi:10.3390/biom11050707)
Supplement: Supplementary file 1 [file biomolecules-11-00707-s001.zip › biomolecules-1178890-SI.pdf]

biomolecules

MDPI

SUPPLEMENTARY MATERIAL

α-Difluoromethylornithine-induced cytostasis is reversed by exogenous polyamines, not by thymidine supplementation

Mervi T. Hyvönen [1*], Maxim Khomutov [2], Jouko Vepsäläinen [1], Alex R. Khomutov [2], Tuomo A. Keinänen [1]

[1] School of Pharmacy, Biocenter Kuopio, University of Eastern Finland, Kuopio Campus, P.O. Box 1627 Kuopio, FI-70211 Finland;

[2] Engelhardt Institute of Molecular Biology, Russian Academy of Sciences, Vavilov Street 32, Moscow 119991, Russia;

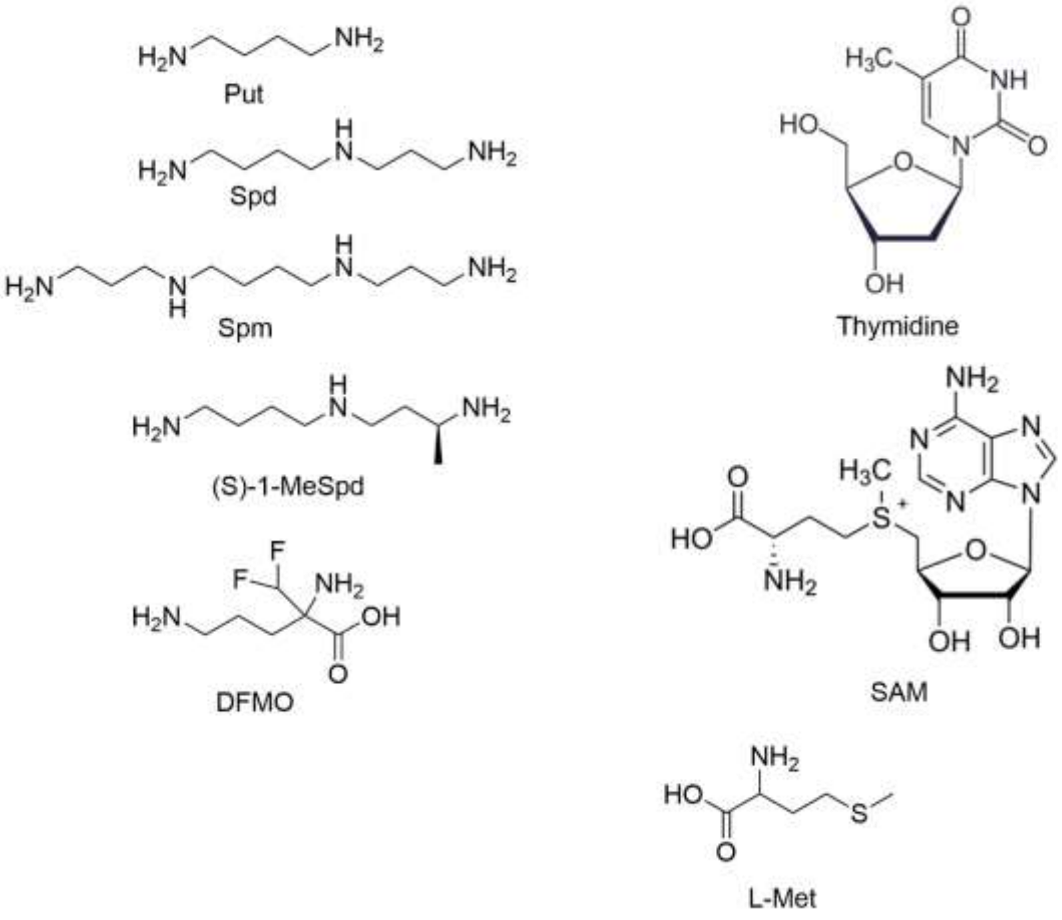

Figure S1. Structures of various small molecules related to this work.

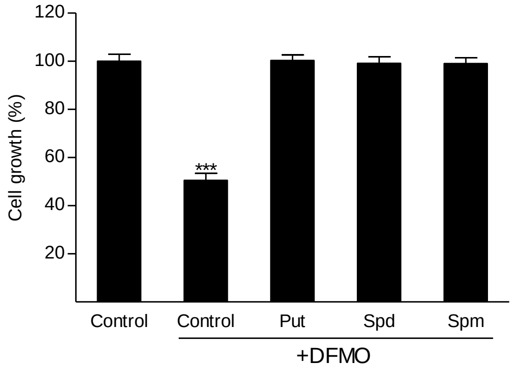

Figure S2. Effect of natural polyamines on growth of DFMO-treated DU145 cells. The cells were treated with 10 μM polyamines in the presence of DFMO (5 mM) for 4 days. All plates also contained 1 mM aminoguanidine to inhibit amine oxidases present in fetal bovine serum. Data are means ± SD, n=6. *** refers to statistical significance of $p<0.001$ as compared to control group.

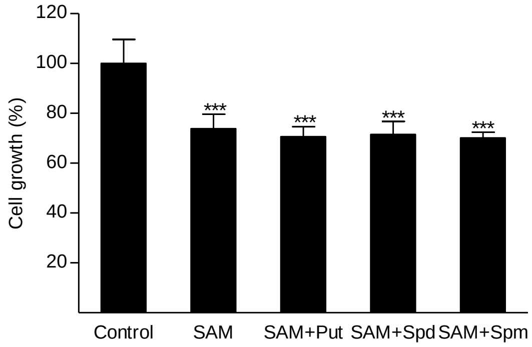

Figure S3. Effect of natural polyamines on growth of SAM-treated DU145 cells. The cells were treated with 10 μM polyamines in the presence of 100 μM SAM for 4 days. All plates also contained 1 mM aminoguanidine to inhibit amine oxidases present in fetal bovine serum. Data are means ± SD, n=6. *** refers to statistical significance of $p<0.001$ as compared to control group.

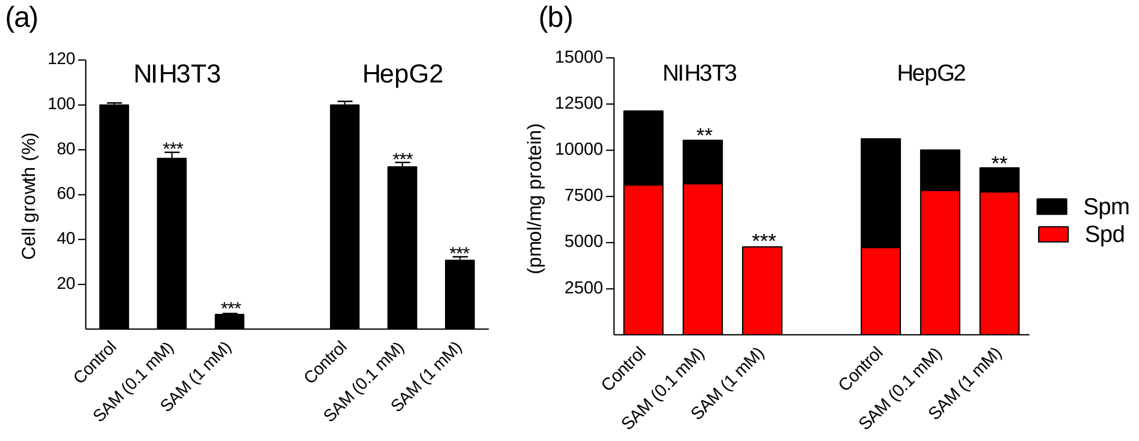

Figure S4. Effect of SAM on the (a) growth and (b) polyamine pools of NIH3T3 and HepG2 cells. The cells were treated with 0.1 mM or 1 mM SAM for 3 days. Data are means ± SD, n=3. ** and *** refer to statistical significance of $p<0.01$ and $p<0.001$, respectively, as compared to control group.
